# Influenza A Virus Cell Entry, Replication, Virion Assembly and Movement

**DOI:** 10.3389/fimmu.2018.01581

**Published:** 2018-07-20

**Authors:** Dan Dou, Rebecca Revol, Henrik Östbye, Hao Wang, Robert Daniels

**Affiliations:** Department of Biochemistry and Biophysics, Stockholm University, Stockholm, Sweden

**Keywords:** influenza A virus, viral ribonucleoprotein, hemagglutinin, viral entry mechanism, viral envelope proteins, HA and NA, viral replication, neuraminidase

## Abstract

Influenza viruses replicate within the nucleus of the host cell. This uncommon RNA virus trait provides influenza with the advantage of access to the nuclear machinery during replication. However, it also increases the complexity of the intracellular trafficking that is required for the viral components to establish a productive infection. The segmentation of the influenza genome makes these additional trafficking requirements especially challenging, as each viral RNA (vRNA) gene segment must navigate the network of cellular membrane barriers during the processes of entry and assembly. To accomplish this goal, influenza A viruses (IAVs) utilize a combination of viral and cellular mechanisms to coordinate the transport of their proteins and the eight vRNA gene segments in and out of the cell. The aim of this review is to present the current mechanistic understanding for how IAVs facilitate cell entry, replication, virion assembly, and intercellular movement, in an effort to highlight some of the unanswered questions regarding the coordination of the IAV infection process.

## Influenza Viruses

**Figure 1:**
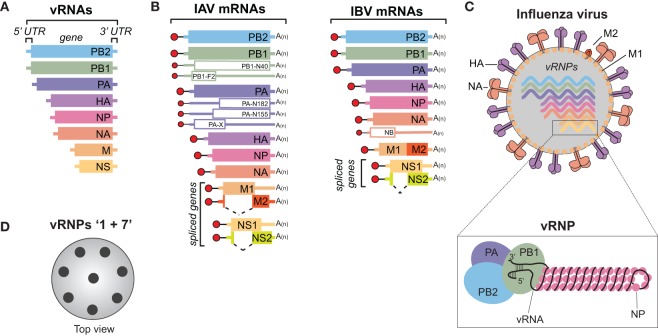
Influenza A and B viruses. **(A)** Schematic of the eight viral RNA (vRNA) gene segments that comprise the influenza A and B genomes. The 5′ and 3′ untranslated regions (UTRs), which contain the viral promoters, are represented with a line, and the box corresponds to the coding region within each vRNA. **(B)** Diagram of the viral mRNAs that are transcribed from the IAV (left) and IBV (right) vRNA templates. Boxes indicate the viral gene product encoded by each mRNA and the dashed lines show the alternative splicing of the IAV M and NS transcripts, as well as the IBV NS transcript. Red circles represent the 5′ M^7^pppG cap, black lines denote the 10–13 nucleotide, host-derived primers that are obtained by the cap-snatching mechanism of the viral polymerase. A(n) corresponds to the 3′ poly-A tail produced by reiterative stuttering of the viral polymerase. The smaller mRNAs (empty boxes) represent transcripts that encode nonessential accessory proteins found in many strains, whereas those that are less prevalent (PB2-S1, M42, and NS3) are not illustrated (6–11). **(C)** Diagram of an influenza A or B virus. The viral membrane proteins HA, NA, and M2 are shown, along with the eight viral ribonucleoproteins (vRNPs), and the matrix protein M1 that supports the viral envelope. To highlight the vRNP components, the illustration beneath the virus is not to scale. A single vRNA gene segment is shown wrapped around multiple nucleoprotein (NP) copies with the conserved promoter regions in the 5′ and 3′ UTRs forming a helical hairpin, which is bound by a single heterotrimeric viral RNA-dependent RNA polymerase (PB1, PB2, and PA). **(D)** Top view of an influenza virus cross-section showing the vRNP “1 + 7” configuration. vRNPs are depicted with black circles as it is not known if the positioning of a particular vRNP is conserved or interchangeable.

Influenza viruses belong to the *Orthomyxoviridae* family and are classified as either type A, B, C, or the recently identified type D (1, 2). Influenza A viruses (IAVs) and type B viruses (IBVs) contain 8, negative-sense, single-stranded viral RNA (vRNA) gene segments (Figure 1A) (3, 4), which encode transcripts for 10 essential viral proteins, as well as several strain-dependent accessory proteins (Figure 1B). In comparison, influenza type C and D viruses only possess seven vRNA gene segments, as the hemagglutinin–esterase fusion protein vRNA replaces the hemagglutinin (HA or H) and the neuraminidase (NA or N) vRNAs (1, 2). IAVs will be the main focus of this review since they are the primary agents responsible for influenza pandemics, and a major contributor to the annual influenza epidemics in the human population (5).

The natural reservoir for IAVs is wild aquatic birds, but they commonly infect other species, including humans, and have even been isolated from penguins in Antarctica (12–15). The ability to adapt to multiple species is a major reason why IAVs are more diverse than IBVs, which are essentially exclusive to humans. Despite the host-range differences, many similarities do exist between these two viruses. Both possess a host-derived lipid membrane, referred to as an envelope, which is decorated on the surface with the viral membrane proteins HA, NA, and to a lesser extent the matrix 2 (M2) protein (Figure 1C) (16–18). The envelope is supported underneath by the matrix 1 (M1) protein, and inside, the eight vRNAs are found as individual viral ribonucleoprotein (vRNP) complexes (Figure 1C, bottom). Each vRNP is comprised of a vRNA that is wrapped around numerous copies of the viral nucleoprotein (NP) and bound by a single copy of the heterotrimeric viral polymerase, consisting of PB1, PB2, and PA (19–21). The polymerase binds the vRNAs at a helical hairpin that results from the base pairing between the conserved semi-complimentary 5′ and 3′ ends (21–23).

Morphologically, IAVs can either form spheres with a diameter of ~100 nm or filaments that can reach up to 20 µm in length [reviewed in Ref. (24)]. However, upon passaging in eggs, or MDCK cells, the filamentous form is generally lost (25, 26). Several studies have attributed the morphology change to M1, presumably through its function in supporting the envelope (27–29). Regardless of the virion shape, HA is the most abundant viral envelope protein, followed by NA, and M2 (30). Recent work has shown that the viral envelope also contains host membrane proteins (30, 31). These proteins are likely recruited based on the lipid composition at the plasma membrane budding site, which can differ between cell types (32, 33). Through possible interactions with each other and M1, the eight vRNPs typicallly form a 1 + 7 configuration inside the virus (Figure 1D) (34, 35). The 1 + 7 configuration may have a mechanistic function, as it is also conserved in type C and D viruses that only possess 7 vRNAs (36). Further supporting the mechanistic concept, it was recently shown that IAVs can package cellular ribosomal RNA (as a vRNP) when one of the vRNAs is made unavailable (37), possibly explaining how type C and D viruses acquire their “eighth” vRNA.

The classification of IAVs into subtypes is based on the genetic and antigenic properties of the surface antigens HA and NA, which mediate viral entry and release, respectively (17, 18). To date, 16 HA (H1-16) and 9 NA subtypes (N1-9) have been found in IAVs isolated from aquatic birds (13). Two additional subtypes for HA (H17 and H18) and NA (N10 and N11) have recently been identified in bats (38, 39), but in contrast to the HA and NA subtypes from the more traditional avian IAVs, these do not appear to recognize sialic acid (SA) (40–42). Despite the numerous possible subtype combinations, only three have consistently persisted in the human population, causing the following pandemics in the process: 1918 and 2009 (H1N1), 1957 (H2N2), and 1968 (H3N2) (43). Currently, only the H1N1 and H3N2 subtypes, as well as the two antigenically distinct IBV lineages (Victoria and Yamagata), are endemic in the human population (44), which is why many IAV vaccines include two representative IAV and IBV strains (5).

A significant challenge in battling IAVs is the constant evolution of the surface antigens (HA and NA) in response to pressure from the host immune system, which is referred to as antigenic drift and antigenic shift. Antigenic drift is most evident in circulating seasonal IAVs, where substitutions by the polymerase that cause mutations in the surface antigen epitopes have continuously been selected to enable reinfection of the same host (45). Antigenic shift is responsible for the development of the IAV pandemics, and it relies on the less frequent process of reassortment, which involves the exchange of vRNAs between two IAVs during co-infection of a cell (43, 46, 47). While reassortment can happen between two related IAVs, antigenic shift occurs when the reassortment process yields a new IAV subtype.

IAVs are also under constant negative selection due to the functional requirements of the viral proteins, and the constraints of the limited genome. Several roles have been reported for most of the IAV proteins. These include the function of HA in receptor binding, as well as membrane fusion, and viral release by the sialidase activity of NA. To perform these functions, the proteins need to correctly fold, oligomerize, and as for the genome itself, they have to be properly trafficked and packaged into new virions. Thus mutations that benefit one property may hinder another. The goal of this review is to highlight these functional requirements by providing a summary of the mechanisms IAVs have evolved to facilitate cell entry, replication, virion assembly and movement, with particular attention to how IAVs coordinate the infection process.

## IAV Cell Binding and Fusion

**Figure 2:**
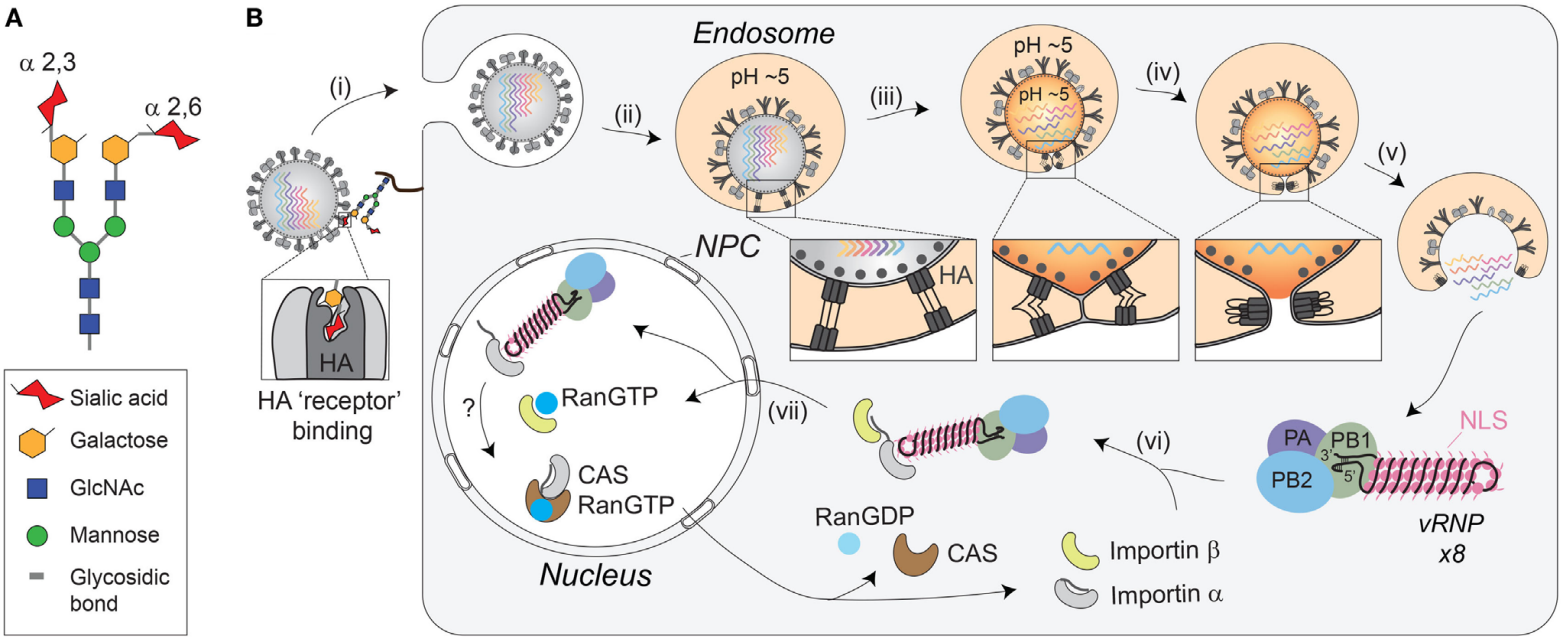
Receptor-mediated cell entry of IAVs. **(A)** Diagram of a bi-antennary N-linked glycan. The terminal sialic acid residues are displayed with an α-2,3 linkage, as well as an α-2,6 linkage, to illustrate the “linear” and “bent” presentations. **(B)** Illustration of IAV cell entry. (i) IAVs initiate cell entry by using the HA receptor-binding domain (located in the HA1 region) to associate with sialylated glycoconjugates on a host “receptor.” Binding to the “receptor” triggers endocytosis. (ii) The virus then traffics to the endosome where the lower pH facilitates a conformational change in HA, exposing the fusion peptide (located in the HA2 region) for insertion into the endosomal membrane. (iii) The HA pre-hairpin conformation begins to collapse, forming a six-helix bundle that promotes hemifusion of the viral envelop with the endosomal membrane. At some point, the M2 channel opens to release the viral ribonucleoproteins (vRNPs) from M1 by acidifying the viral interior. (iv) HA further collapses into a trimer of hairpins to promote the formation of the fusion pore, which (v) releases the vRNPs into the cytosol. (vi) The exposed nuclear localization signals (NLS) on the vRNPs are recognized by the adaptor protein importin-α, leading to the recruitment of importin-β that (vii) facilitates the transport through the nuclear pore complex (NPC) and into the nucleus.

IAVs initiate the infection process by using the HA molecules on the viral envelope. Upon reaching a potential host cell, the HA receptor-binding site attaches the virus to surface glycoconjugates that contain terminal SA residues (Figure 2A) (18, 48, 49). IAVs then scan the cell surface for the proper sialylated “receptor” by using the sialidase function of NA to remove local SAs and liberate nonproductive HA associations (50). Currently, the “receptor’s” identity remains unknown, but it is generally thought that HAs from avian IAVs have higher specificity for receptors with α-2,3-linked SAs that have a “linear” presentation (51, 52), whereas HAs from human IAVs prefer an α-2,6 linkage, which results in a more “bent” presentation (Figure 2A) (53, 54). While these preferences correlate with SA linkages in the respective hosts (55), several studies have shown that matching HA receptor binding preferences with the SA linkages in a particular host is not essential for infection, but is more critical for transmission (56–59). This implies that the IAV “receptor” either displays significant cell tropism in the airways or that IAVs can potentially use more than one receptor.

Despite the unknown identity of the receptor, it is clear that HA-mediated binding to the receptor triggers endocytosis of the virion (Figure 2B, step i). The endocytosis can either occur in a clathrin-dependent manner, involving dynamin and the adaptor protein Epsin-1 (60–62), or by macropinocytosis (61, 63, 64). Once inside the cell, the virus is trafficked to the endosome, where the low pH activates the M2 ion channel (61, 65, 66), and causes a large conformational change in HA that exposes the fusion peptide (Figure 2B, step ii) (67–69). Opening of the M2 ion channel acidifies the inside of the viral particle, releasing the packaged vRNPs from M1 (Figure 2B, step iii), which enables the transfer of the vRNPs to the host cytoplasm following HA-mediated fusion (70, 71).

Fusion of the viral-endosomal membranes by HA occurs through multiple steps [reviewed in Refs. (72, 73), and requires cleavage of HA by host cell proteases into two subunits, HA1 and HA2 (55, 74, 75)]. The cleavage (see HA Proteolytic Activation at the *Golgi* or Plasma Membrane) is required to enable the exposure of the fusion peptide on the N-terminus of the HA2 upon the pH change in the endosome (69). Once exposed, the fusion peptide inserts into the endosomal membrane, while the C-terminal transmembrane domain (TMD) anchors HA2 in the viral membrane, creating a pre-hairpin conformation (see Figure 2B, step ii “box”). The HA2 trimers then fold back on themselves creating a hairpin that begins to position the two membranes in close proximity to each other (see Figure 2B, step iii “box”). The hairpin bundles then further collapse into a six-helix bundle, and in doing so, the two membranes come closer together enabling the formation of the lipid stalk, and the subsequent fusion of the two membranes (Figure 2B, step iv). To date, not all of these stages have been observed with HA and some have been inferred based on observations of related fusogens from other viruses.

## IAV Genome Trafficking to the Host Cell Nucleus

In contrast to the early steps in IAV entry, vRNP trafficking to the nucleus following the fusion event is highly dependent on the host cell machinery and transport pathways [reviewed in Ref. (76)]. Supported by numerous studies, the current model is that the newly released cytoplasmic vRNPs use the importin-α–importin-β nuclear import pathway to gain entry to the host cell nucleoplasm (Figure 2B, steps vi and vii) (77–83). To initially engage this pathway, it is thought that the vRNPs use the surface exposed nuclear localization sequences from the numerous NP molecules to recruit the adapter protein importin-α (80–82). Upon binding to the vRNP, importin-α is recognized by the importin-β transport receptor, which directs the vRNP to the nuclear pore complex, where it is transported into the nucleoplasm.

Recent improvements in imaging and RNA labeling techniques have made it possible to monitor the entire entry process in single cells (61, 62, 83–85). The cumulative results from these studies show that IAVs can deliver their vRNPs from the cell surface to the nucleus in approximately 1 h, with entry and fusion occurring rather quickly (~10 min), and nuclear import requiring the bulk of the time (85). A striking observation from these studies is the efficiency with which the eight vRNAs reach the nucleus, indicating how effectively vRNPs recruit the host nuclear import factors. Supporting this observation, it was shown that NP adaptation to the importin-α isoforms of a particular species is crucial for productive IAV infections (86). While the bulk of the vRNP trafficking work has been carried out using various immortalized cell lines, the potential species related differences, and the essential role of vRNP trafficking in reassortment, emphasize the need for further methodology development to examine the details of IAV entry in primary cells and tissue explants.

## Replication of the vRNAs

**Figure 3:**
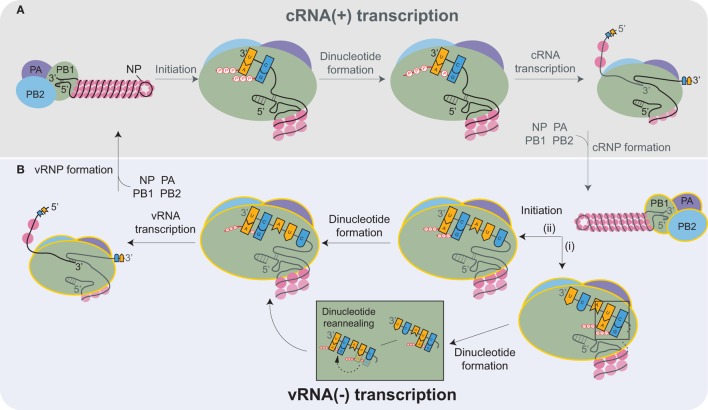
Transcription of the complimentary RNA (cRNA) and viral RNA (vRNA) by the heterotrimeric viral RNA-dependent RNA polymerase (PB2, PB1, and PA). **(A)** The viral polymerase initiates transcription of the positive-sense cRNA upon base-pairing of ATP and GTP with the complimentary nucleotides in the 3′ end of the vRNA. The subsequent formation of the A-G dinucleotide is followed by elongation of the cRNA transcript. Nucleoprotein (NP) molecules successively bind to the cRNA as it exits the polymerase, promoting cRNP assembly. cRNP formation is completed upon the termination of transcription and with the binding of a newly synthesized viral polymerase (yellow outline). **(B)** vRNA transcription proceeds in a similar manner as cRNA synthesis. Recent structures support a model where (i) ATP and GTP base pair to the nucleotides located 4 and 5 bases from the cRNA 3′ end, and there form a dinucleotide, which then disassociates and reanneals with the bases at positions 1 and 2. (ii) Alternatively, ATP and GTP could bind directly to the terminal nucleotides and form a dinucleotide. Both mechanisms would position the dinucleotide at the cRNA 3′ end, which is necessary to transcribe a full-length vRNA. Similar to cRNP formation, multiple NPs and a viral polymerase bind to the newly transcribed vRNA to produce a new viral ribonucleoprotein (vRNP).

Inside the nucleus, the heterotrimeric viral RNA-dependent RNA polymerase carries out the transcription and replication of the vRNAs [reviewed in Refs. (87, 88)]. The replication of the influenza genome involves two steps: transcription of complimentary RNA (cRNA), followed by transcription of new vRNA copies using the cRNAs as templates. The cRNAs are produced by an unprimed process that relies on the correct complementation of free rNTPs (generally GTP and ATP) with the 3′ end of the vRNA template (Figure 3A) (89, 90). The nucleotide complementation locks the vRNA template into the polymerase active site within the PB1 subunit and results in the formation of an A–G dinucleotide from which the cRNA is elongated (91). Upon exiting the polymerase, the cRNA associates with newly synthesized NP molecules and a single copy of the viral polymerase to assemble into a cRNP (90).

Currently, it is thought that the newly produced viral polymerases, which are incorporated into the cRNPs, generate multiple vRNA copies in a manner similar to cRNA transcription (Figure 3B). However, there is one distinction related to the difference in the positioning of the longer 3′ end of the positive-sense cRNA. Due to the increased length, the cRNA is positioned in the polymerase such that the rNTP annealing and dinucleotide formation is likely to occur at the nucleotides located 4 and 5 bases from the cRNA 3′ end (Figure 3B, pathway i) (90, 92–94). The dinucleotide primer then has to dissociate and reanneal to the nucleotides at the 3′ end prior to elongation (Figure 3B). Alternatively, the cRNA 3′ end could reposition within the polymerase due to rNTP binding, resulting in the generation of full-length vRNA transcripts directly (Figure 3B, pathway ii). The transient nature of the rNTP annealing and dinucleotide formation makes it technically challenging to exclude either possibility. The remaining task of assembling a vRNP is analogous to cRNP formation.

## Viral mRNA Transcription

**Figure 4:**
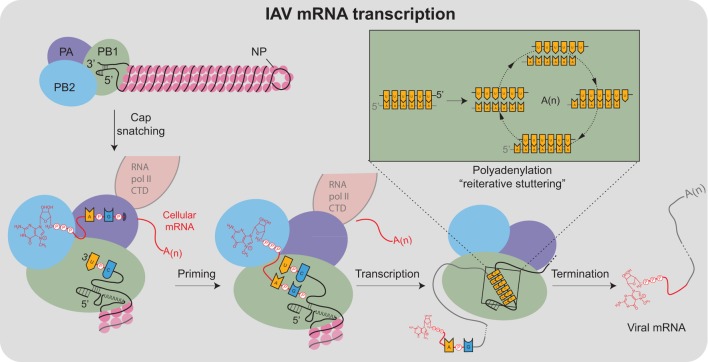
Transcription of IAV mRNAs by the viral polymerase. Viral mRNA transcription occurs when the viral ribonucleoproteins reach the host cell nucleus and is assisted by the association of the viral polymerase (PA subunit) with the cellular RNA polymerase II C-terminal domain (RNA pol II CTD). Transcription initiates by a “cap-snatching” mechanism where the PB2 subunit binds to the 5′ cap of a host mRNA (red). Cap binding positions the region of the mRNA 10–13 nucleotides downstream for cleavage by the endonuclease domain in the PA subunit. Following cleavage, a conformational shift repositions the acquired mRNA capped primer to the PB1 subunit where the 3′ end base-pairs with a complimentary sequence at the vRNA 3′ end. Following the priming event, the viral polymerase extends the mRNA transcript. The transcription is terminated by a “reiterative stuttering” process (depicted in the box), which occurs when the polymerase encounters the 5–7 consecutive uracil bases at the vRNA 5′ end. The “reiterative stuttering” function likely involves multiple cycles of dissociation and reannealing, and effectively polyadenylates [A(n)] the viral mRNA by continuously repositioning the elongating 3′ end on the uracil-rich region of the vRNA template.

Viral mRNA transcription from the vRNA templates is primed, making it significantly more efficient than cRNA and vRNA transcription (95). The viral polymerase obtains the primers through a mechanism termed cap snatching (96), which is aided by the association with the cellular RNA polymerase II C-terminal domain (Figure 4) (97–99). For cap snatching, the viral polymerase uses the PB2 subunit to bind to 5′ caps of nascent host transcripts (100) and the PA subunit endonuclease domain to cleave 10–13 nucleotides downstream of the 5′ cap (101–103). The PB2 cap-binding domain then rotates to position the newly acquired capped primer into the PB1 catalytic center where it is extended using the vRNA as a template (95). Finally, each transcript is polyadenylated through a reiterative stuttering′ process, which occurs when the polymerase encounters the short poly-U sequence at the vRNA 5′ end (Figure 4 “box”)(104, 105). This process likely involves multiple cycles of dissociation, repositioning, and reannealing of the mRNA to this template region of the vRNA to achieve polyadenylation.

During the course of infection, mRNA synthesis occurs before cRNA and vRNA transcription, and mRNA transcription is much more abundant because the use of primers significantly increases the initiation efficiency (106). The initial mRNAs are transcribed by the vRNP-associated polymerases and exported from the nucleus for translation by cytoplasmic ribosomes (93). However, the M and NS transcripts also possess donor and acceptor splice sites that match well with those in human transcripts (107). These sites recruit the cell spliceosome, which produces the spliced transcripts that encode for the M2 and NS2 proteins, respectively (108–112). The NS transcript has been reported to maintain a similar ratio of non-spliced and spliced transcripts throughout infection (113), whereas the ratio of the spliced M transcripts (encoding M2) have been shown to increase during infection (114). These observations imply that NS1 and NS2 are always equally expressed, while M2 expression is more biased toward the later stages of infection. However, it is likely that the splicing efficiency of the NS and M transcripts differs between IAV strains (115, 116).

## Assembly and Trafficking of vRNPs

**Figure 5:**
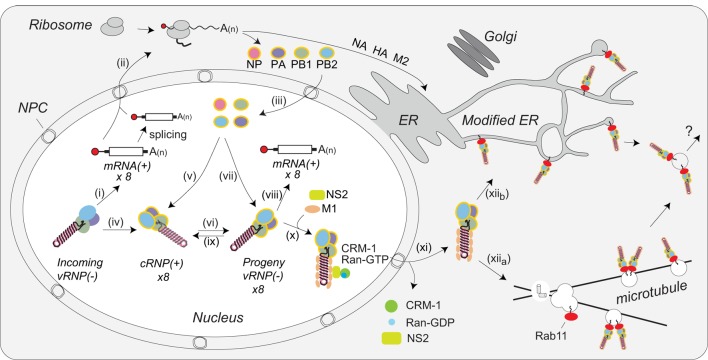
Coordination of viral ribonucleoprotein (vRNP) assembly and trafficking to the plasma membrane. Upon entry into the host cell nucleus, (i) the vRNP-associated viral polymerase transcribes the viral mRNAs. (ii) The mRNAs are either directly, or after alternative splicing, exported for translation by cytosolic ribosomes. (iii) Newly synthesized viral polymerase subunits (PA, PB1, and PB2) and nucleoprotein (NP) are imported back into the nucleus. (iv) Due to the inefficient dinucleotide priming, the vRNP-associated viral polymerase also infrequently transcribes complimentary RNA (cRNA) copies that assemble into cRNPs *via* (v) binding of a newly synthesized viral polymerase (PA, PB1, and PB2) and NP. (vi) The polymerase transcribes viral RNA (vRNA) copies from the positive strand in the cRNPs and these assemble into vRNPs by (vii) association with a new viral polymerase (PA, PB1, and PB2) and NP. Once assembled, the new vRNPs can (viii) transcribe additional viral mRNAs, (ix) transcribe new cRNA copies, or (x) associate with the newly synthesized viral proteins M1 and NS2 to facilitate the recruitment of CRM1, which (xi) mediates the nuclear export of the vRNP. (xii_a_) Once exported, the vRNPs then associate with Rab11 that assists in the trafficking of the vRNPs toward the cell surface. The vRNP trafficking either occurs by Rab11-containing vesicles associated with microtubules or (xii_b_) through Rab11 located in the modified endoplasmic reticulum (ER) membranes. How the vRNPs reach the budding site at the plasma membrane is currently not known.

IAV protein synthesis is entirely dependent on the translation machinery of the host cell. Following nuclear export [reviewed in Ref. (117)], the translation of the viral mRNAs is divided between cytosolic ribosomes (for PB1, PB2, PA, NP, NS1, NS2, and M1) and endoplasmic reticulum (ER)-associated ribosomes for the membrane proteins HA, NA, and M2 (Figure 5, steps i and ii). Nuclear localization sequences on the newly synthesized NP proteins and polymerase subunits (PB1, PB2, and PA) target these proteins into the nucleus by recruiting the importin-α-importin-β pathway that is utilized for vRNP nuclear import (Figure 5, step iii). The NP and PB2 proteins are imported individually, whereas the PB1 and PA proteins are imported as a heterodimer (81, 118). In the nucleus, these newly synthesized proteins assist in viral mRNA transcription and vRNA replication. NP monomers bind to 12 nucleotide stretches with a partial G bias in vRNAs, and presumably cRNAs, to assemble vRNPs and cRNPs through a process that may be regulated by the NP phosphorylation (Figure 5, steps v and vii) (119–121). The heterotrimeric polymerase assembles and binds to the newly formed cRNPs to transcribe vRNAs (Figure 5, step vi) that upon formation into vRNPs can generate additional viral mRNA (Figure 5, step viii), or cRNA transcripts (Figure 5, step ix) (90, 93).

The viral RNA-binding protein NS1 is synthesized early and also imported into the nucleus, where it can act as an inhibitor of interferon signaling [reviewed in Ref. (122)]. In addition, NS1 may contribute to viral mRNA export from the nucleus by linking the viral transcripts to the cellular nuclear export components TAP/NXF1, p15, Rae1, E1B–AP5, and the nucleoporin NUP98 (123). NS2 (alternatively known as the nuclear export protein) and M1 are imported into the nucleus as well. Multiple studies have implicated these two proteins in the nuclear export of vRNPs (70, 71, 124–127). While the mechanism remains unclear, current data support a model where M1 acts as an adaptor protein linking NS2 to vRNPs (Figure 5, step x) (128, 129). Through established interactions with CRM1, NS2 is then able to target the vRNP complex to the CRM1 nuclear export pathway for transport to the cytoplasm (127), where M1 potentially prevents the re-import of vRNPs by blocking access to the NP nuclear localization sequences (Figure 5, step xi) (71).

Within the cytoplasm the vRNPs are trafficked toward the plasma membrane for viral assembly by Rab11. Rab11 facilitates the interaction by associating with the viral polymerase PB2 subunit (130), potentially providing a quality control mechanism that ensures new virions incorporate vRNPs carrying a polymerase. Earlier studies proposed that vRNPs specifically associate with Rab11 on recycling endosomes, which use microtubules for transport toward the cell surface (Figure 5, step xii_a_) (130–132). An alternative model has recently been proposed where infection causes tubulation of the ER membrane network and the vRNPs bind to Rab11 molecules that have localized to this network for trafficking toward the plasma membrane (Figure 5, step xii_b_) (133). Currently, it is not known how vRNPs are transferred to the plasma membrane in either model, or how IAVs incorporate all eight of the different vRNPs in a “1 + 7” configuration. While several studies have indicated that specific vRNP associations likely contribute to the packaging of the eight vRNPs (35, 134, 135), the underlying mechanisms remain to be established.

## ER Targeting and Maturation of the IAV Membrane Proteins

**Figure 6:**
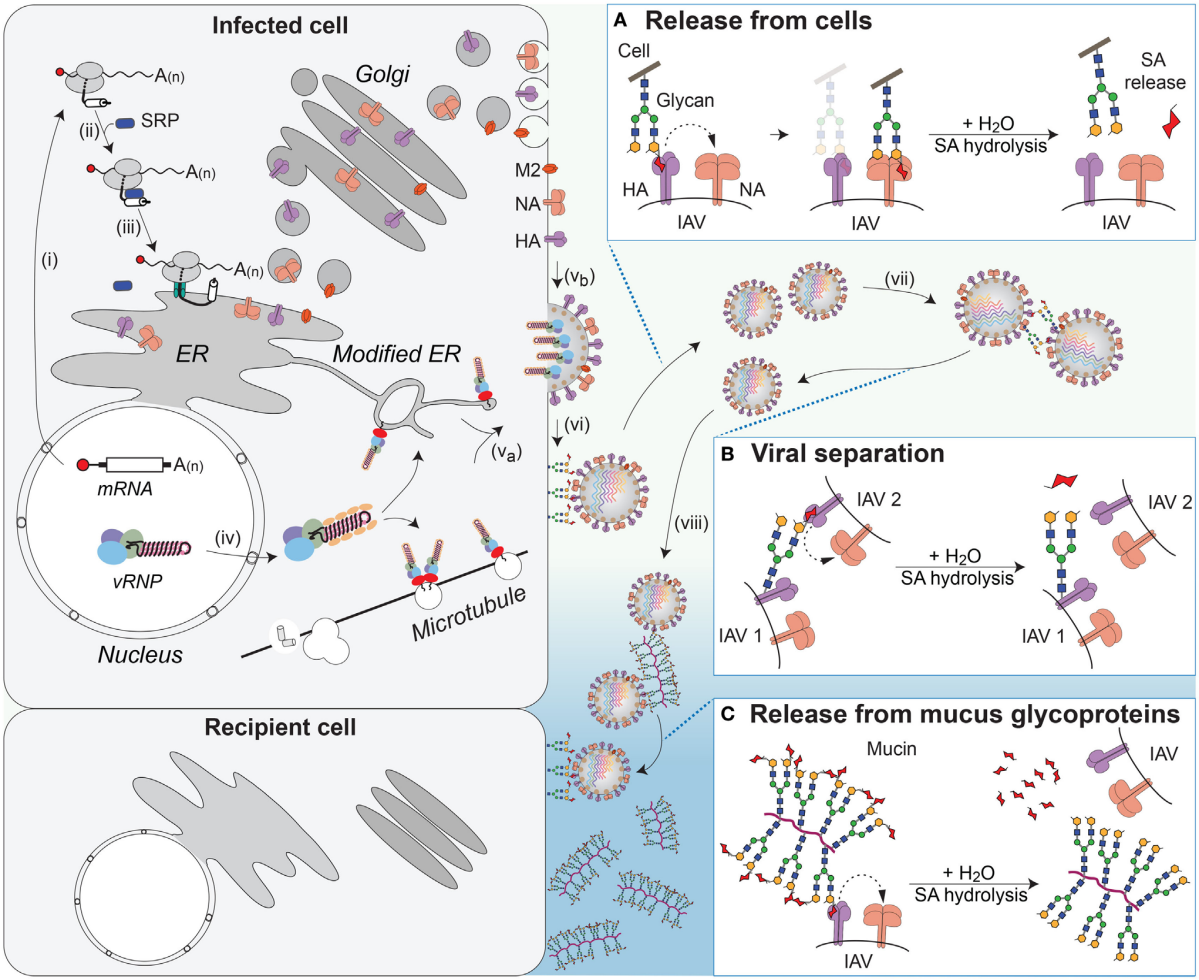
NA contributions to viral release and intercellular movement. (i) Viral mRNAs encoding the membrane proteins NA, HA, and M2 are exported for translation by cytosolic ribosomes. (ii) Exposure of the N-terminal signal sequence (HA) or transmembrane domains (NA and M2) recruits the signal recognition particle (SRP), which (iii) targets the ribosome–nascent chain complex for synthesis at the endoplasmic reticulum (ER). Following synthesis, the proteins oligomerize and are trafficked through the *Golgi* to the plasma membrane. (iv) Late in replication, the viral ribonucleoproteins (vRNPs) are exported from the nucleus and (v_a_) trafficked to the budding regions in the plasma membrane, where (v_b_) HA and NA have co-localized, with M2 at the budding boundary. (vi) Following budding, progeny virus can remain associated with the infected cell’s surface through HA binding to sialic acid (SA). (Box A) The envelope protein NA promotes release of the virus from the infected cell surface by hydrolyzing the glycosidic bond attaching the SAs. (vii) SAs present on the glycans of HA and NA can result in HA-mediated virus–virus association. (Box B) NA can separate the viruses by removing these SAs. (viii) In the respiratory tract, the epithelium is protected by mucus, rich in sialylated glycoproteins such as mucin, which can associate with HA and slow viral movement. (Box C) NA can cleave off the SAs from the glycoproteins within the mucus to facilitate movement of the virus to neighboring cells.

The IAV membrane proteins, which are ultimately destined for the viral envelope, are synthesized by ribosomes associated with the ER membrane. Similar to cellular secretory proteins, ribosome–nascent chain complexes containing NA, HA, or M2 are co-translationally directed to the ER by interactions of their hydrophobic targeting sequences with the signal recognition particle (SRP) (Figure 6, step ii) (136–139). The cleavable signal sequence on HA facilitates the interaction with SRP, whereas NA and M2 use their respective TMD as an ER targeting sequence. Once bound, SRP targets the ribosome–nascent chain complexes to the SRP receptor in the ER membrane (Figure 6, step iii), which transfers the ribosome to a Sec61 protein-conducting channel known as the translocon (140–142). Linked to the dependence on SRP, mutations that alter the targeting sequence hydrophobicity of cellular secretory proteins have been shown to decrease their ER targeting and subsequent synthesis (143, 144). Although this aspect has not been examined for the IAV membrane proteins, there is evidence that the hydrophobicity of their ER-targeting sequences change (138, 148), which suggests IAVs potentially use this mechanism to titrate NA and HA expression.

The translocon enables passage of the elongating NA, HA, and M2 polypeptides into the ER lumen and facilitates the membrane partitioning of their respective TMD segments through a lateral gate (145, 146). To activate the membrane integration, the TMD segments have to be of the appropriate length and hydrophobicity (146, 147). In human H1N1 and H3N2 viruses, these criteria are conserved in the TMDs of HA and M2, but not in the TMD of NA, as it has become progressively less hydrophobic in the H1N1 viruses (148). The uncharacteristic hydrophobicity loss was shown to be possible because of the NA TMD being positioned at the N-terminus (138). The positioning (~435 amino acids from the C-terminus), combined with the slow rate of ribosomal translation (~5 amino acids per second), likely provides these nontypical TMDs with significant time to properly orientate and facilitate membrane insertion during the co-translational translocation process.

During translocation, the N-terminus of HA and M2 is directly translocated into the ER lumen, whereas NA inverts, positioning the C-terminus in the ER lumen (137, 138). In addition, HA and NA receive multiple N-linked glycans. The glycans are transferred by the oligosaccharyltransferase to Asn–X–Ser/Thr sequences, and vary in number as well as positioning based on the strain, or subtype (149). One function of the glycans is to increase the folding efficiency of NA and HA by recruiting the lectin chaperones (calnexin and calreticulin) and the associated oxidoreductase ERp57, which aids in disulfide bond formation (136, 150–152). This is especially crucial for the HA and NA proteins that possess a significant number of intramolecular disulfide bonds (e.g., six in HAs, eight in N1, and nine in N2) (153–155). By contrast, M2 possesses two intermolecular disulfide bonds in its tetrameric conformation (156). Depending on the subtype, NA tetramers also possess 2 or more intermolecular disulfide bonds.

Oligomerization of HA involves the trimerization of independently folded monomers, whereas NA tetramerization has been proposed to result from the pairing of two co-translationally formed dimers, which assemble through a process involving the N-terminal TMD of NA (150, 157). In line with this model, it has been shown that the TMD is essential for proper NA folding, and that the decreasing hydrophobicity in the N1 TMDs functions to support the folding and oligomerization of the enzymatic head domain (158, 159). IAVs easily achieve the protein concentration-dependent requirement for oligomerization due to the abundance of HA and NA that is synthesized during an infection. However, these high synthesis levels at the ER can also be deleterious by activating the ER-stress response. Indeed, several studies have shown that IAV replication does activate the ER-stress induced unfolded protein response (160, 161), but this response is also mitigated by the inhibition of the eIF2α-kinase and stress granule formation through the functions of other viral proteins (162).

Despite everything that is known about the synthesis and assembly of the IAV membrane proteins, several aspects have yet to be addressed. These include obtaining atomic structures of full-length HA and NA in a membrane, something that should become easier to address with the advances in cryo-electron microscopy structure determination. Identifying if the NA protein removes SA residues directly from substrates within the *Golgi*, as this could decrease the effectivity of nonmembrane permeable NA inhibitors. It is also unclear how IAVs regulate the timing and expression levels of the viral proteins as viral mRNA transcription shows little temporal variation (163, 164). While it is likely that M2 is regulated in part by splicing (112, 114), this does not apply to HA and NA. Recent work has linked NA and HA regulation to the nucleotide composition of the 5′coding regions for their ER-targeting sequences, which dramatically differ from the profile of corresponding regions in human secretory protein mRNAs (165, 166). An obvious candidate for post-transcriptional regulation is the viral RNA-binding protein NS1. Indeed, many studies have shown that NS1 can increase translation of particular mRNAs, possibly by enhancing the translation initiation rate through the recruitment of eIF-4G to the 5′region of viral mRNAs (165, 167–171). However, a clear mechanistic picture for influenza protein regulation is lacking.

## HA Proteolytic Activation at the *Golgi* or Plasma Membrane

HA traffics from the ER as a fusion incompetent precursor termed HA0. To gain its fusion function, HA must be cleaved into the subunits HA1 and HA2 (74, 172, 173). The cleavage occurs in either a monobasic, or a multibasic, cleavage site (55). Multibasic sites are commonly found in highly pathogenic avian IAVs and are cleaved by furin, a calcium-dependent serine endoprotease that is located within the *trans-Golgi* network (174). Furin is also ubiquitously expressed (175), which is one of the major reasons why avian IAVs with a multibasic cleavage site are generally more pathogenic.

By contrast, human (and low pathogenic avian) IAVs encode for HAs with a monobasic cleavage site, which have been shown to be processed by different proteases in human respiratory epithelial cells. These include the transmembrane protease serine S-1 member 2 (TMPRSS2), human airway trypsin-like protease (HAT), and possibly TMPRSS4 (176, 177). HAT localizes at the plasma membrane where it can either cleave newly synthesized HA or the HA found in cell-associated virions (178, 179). Similar to furin, TMPRSS2 resides in the *trans-Golgi* network, where it cleaves HA en route to the plasma membrane. The M2 ion channel is thought to prevent the premature activation of HA following cleavage by equilibrating the slightly acidic pH of the *Golgi* (180, 181). Distinct from furin, TMPRSS2 expression has been found to be more restricted to the upper and lower respiratory tract, whereas HAT was mainly shown to be expressed in the upper respiratory tract (182). These cell tropisms suggest that lower respiratory infections are likely mediated by TMPRSS2, and could be one of the primary reasons human IAVs are confined to the epithelial layer of the respiratory tract.

## IAV Assembly and Budding

Compared with the bulk lipid profile of the plasma membrane, IAV envelopes are enriched in cholesterol and sphingolipids (32), indicating that they bud from distinct apical plasma membrane regions often referred to as “rafts” (183). However, infectious IAVs must possess mechanisms to target the eight vRNPs, M1, HA, NA, and M2 to these sites in the membrane (184, 185). HA is believed to localize to these distinct regions based on fatty acid modifications of the C-terminal cysteine that occur in the *Golgi* (186–189), whereas NA enrichment has previously been attributed to a property in the C-terminus of the TMD (190). In contrast, M2 has been shown to accumulate at the boundaries of these budding domains (191), and the cytosolic protein M1 has been proposed to localize to the budding region by associating with the short cytoplasmic tails of HA and NA (192). However, it is equally plausible that NA and HA create membrane domains with a unique lipid profile that have a high affinity for M1. Finally, the vRNPs, delivered to the cell periphery by Rab11, are thought to localize to the budding site by binding to M1 (193, 194).

In addition to orchestrating the assembly of the correct viral components at the apical budding site, IAVs also have to remodel the membrane to induce bud formation, and ultimately scission of the viral envelope from the plasma membrane. To promote bud formation, the virus must first induce significant curvature in the membrane and then constrict the two opposing membranes of the viral envelope to help to facilitate membrane scission. Curvature can be induced by (i) protein or “molecular” crowding on one leaflet of a bilayer, (ii) association of curved or “bending” proteins with the bilayer, (iii) biased accumulation of cone shaped lipids in one leaflet of the bilayer, or (iv) the cytoskeleton (195). Based on cumulative data regarding budding, IAVs appear to induce membrane curvature through a combination of these mechanisms. Indicative of using molecular crowding and bending proteins, several studies have demonstrated that HA and NA expression is sufficient to induce budding, and that the efficiency and shape uniformity benefit from the presence of M1 (196–199). These results indicate that the abundance of HA and NA on one side of the membrane can contribute to curvature. It also is intriguing to speculate that the asymmetric (154) shape of NA plays a role in this process as it is often seen clustering in the viral membrane (16, 199). By contrast, M1 appears to be analogous to a membrane-bending protein as it recruited to the cytosolic side of the membrane budding site, oligomerizes upon reaching the membrane, and these oligomers have been modeled to form curved structures (200–202). Based on these properties, it is plausible that M1 significantly influences the membrane curvature at the budding site, potentially explaining its role in discerning whether IAVs form spheres or filaments (27, 203).

The ion channel M2 localizes to the budding site boundary and has also been shown to contribute to IAV scission by functioning as a membrane-bending protein (191, 204). The membrane-bending property of M2 is localized in an amphiphilic α-helix that can incorporate the amino acid side chains from its hydrophobic face into a leaflet of the bilayer. With this domain positioned in the cytosol, the intercalation results in negative membrane curvature, which has been proposed to facilitate viral bud neck formation and scission, presumably by decreasing the distance between the two opposing membranes of the viral envelope (204). While much of the framework concerning IAV budding has been established, it has been difficult to identify the details of the budding process, in part due to the mobility and heterogeneity of the plasma membrane. The lack of strong phenotypes from domains proposed to contribute to budding could also imply that IAVs have built redundancy into the budding process (205–207). The possibility of redundancy is certainly plausible, as IAVs contain the necessary components to allow for a combination of lipid recruitment, molecular crowding, and a membrane-bending protein.

## IAV Cell Release and Movement

Once the newly assembled IAVs bud, their release is highly dependent on the sialidase activity of NA. NA is a homotetramer, and each subunit is comprised of a short N-terminal cytoplasmic tail (six amino acids), followed by a TMD, a length variable stalk, and a globular enzymatic head domain (208). The globular head domain forms a 6-bladed propeller structure, where each blade is comprised of four antiparallel β-sheets that are stabilized by disulfide bonds (155, 209, 210). The catalytic Tyr residue is found in a highly conserved active site that forms a deep pocket in the center of each monomer (211). All of the residues necessary for catalysis exist within each monomer (212), which has made it difficult to reconcile why NA evolved to function as a tetramer (208, 213, 214). Structures of the enzymatic head domain indicate that NA tetramers bind up to five calcium ions and calcium has been shown to contribute to NA activity (155, 208, 215). However, it remains unclear why influenza NA has evolved to position a calcium ion at the tetrameric interface.

NA facilitates viral release by catalyzing the hydrolysis of the glycosidic linkage that attaches SA to underlying sugar molecules (216–218). By removing local SA residues, NA prevents HA binding at the cell surface, which facilitates the release of the virus during budding (Figure 6A and step vi) (219, 220). NA has also been shown to promote the separation of IAVs by removing SA residues from the N-linked glycans located on the HA and NA molecules in the viral envelope (Figure 6B and step vii) (221). In contrast to HA, NAs from human IAVs show a general preference for α2,3-linked SA with variable abilities to cleave α2,6-linked SA residues (208, 222, 223). However, a thorough analysis of NA SA preference is lacking. More recent studies have found that some strains possess NAs that are inefficient enzymes, but still capable of SA binding, raising the question of whether a poor NA enzyme could contribute to, or replace, the HA receptor-binding function (224, 225).

The movement of IAVs from cell to cell in the respiratory epithelium is significantly different from that in immortalized cell lines grown in liquid culture due to the presence of different cell types and a mucus layer. The mucus layer provides a protective barrier for the epithelium and is rich in heavily glycosylated mucins that can interact with IAVs and limit cell binding (226, 227). Studies measuring viral movement through mucus and respiratory epithelial cells have shown that NA-mediated cleavage of SAs from mucins enhances IAV movement through the mucus layer and infectivity (Figure 6C and step viii) (226, 228, 229). Recent work showed that this function may also apply to transmission, as IAVs that possess low NA activity, and are inhibited by mucus, are deficient in aerosol and contact transmission (230).

## Perspectives

IAVs are constantly exposed to negative and positive selection pressure, which shapes how the virus evolves. The functional requirements of each IAV protein, such as enzyme catalysis, substrate binding, oligomerization, and domains that perform essential interactions with host proteins all combine to create substantial negative selection pressure that often manifests in the form of sequence conservation. Negative pressure can also come from functions within the vRNA sequences. These include promoters and “packaging signals,” but are also likely to involve aspects such as the formation of structural elements, or possibly mediating vRNP interactions that generate the 1 + 7 assembly in viral particles. In addition, the exposure of IAVs to the immune response and constantly changing environments such as host, temperature, pH, cell type, and antivirals result in positive selection pressure. Experimentally, addressing each type of selection has its caveats, but clearly a holistic picture of both IAV and host functions are required to begin predictions of evolutionary constraints on the virus.

Most studies on the influenza evolutionary process focus primarily on antigenic drift and antigenic shift. However, all the viral transcribed RNAs are subject to replication errors by the viral polymerase, which are estimated at 1 per 2,000–10,000 nucleotides (231–233). Consequently, both the viruses and the viral proteins are likely to exist as large heterogeneous populations during an infection. As many IAV proteins are homo-oligomers this can potentially generate heterogeneity within individual protein complexes that could have functional advantages. By applying single particle and single cell analysis, these types of aspects are beginning to be investigated (234). Another interesting approach is deep mutational scanning, which has been used to examine the site-specific amino acid tolerance of IAV proteins in general, and in the context of different selection pressure (235–238).

Currently, the best characterized protein in IAVs is HA, which has two primary functions, (i) to initiate binding to the host cell and (ii) to deliver the vRNPs to the host cell cytosol by fusing the viral and endosomal membranes. These functions are efficiently divided between the two domains of HA (HA1 and HA2), created by proteolysis. The receptor-binding site responsible for entry is located in the considerably larger HA1 subunit that is known to be immunodominant, explaining the high sequence variability in this region (239). By contrast, the smaller HA2 subunit, containing the fusion peptide that is necessary to deliver the viral genome to the host cell, shows considerably higher sequence conservation. This organization is logical from the viral perspective as the large HA1 subunit likely blocks antibody recognition of HA2. The viral downside is the need to escape antibodies that inhibit the receptor-binding pocket without losing specificity and the binding function.

Based on this knowledge, several exciting new strategies are being developed to elicit the production of antibodies that target the more conserved region of HA (240–242). The hope is that these strategies will generate broadly neutralizing antibodies that recognize multiple HA subtypes from IAVs and the distinct lineages in IBVs, providing longer lasting immunity and alleviating the threat of potential pandemics. A similar approach using NA would likely provide additional benefits. However, our knowledge of NA lags behind HA. Currently, it is still not known why NA has evolved to function as a tetramer, which is relevant because this property presumably restricts the potential antigenic drift (mutations) it can accommodate and still function.

A relatively overlooked feature in the replication process is the contributions of host RNA-binding proteins (RBPs). Human cells are predicted to encode over 1,500 RBPs, 700 of which are predicted to interact with mRNAs (243). As a RNA virus, it is highly likely that IAVs have evolved to utilize this enormous network of RBPs, which is supported by observations that some RBPs inhibit IAV replication, whereas others contribute (244–246). It should also be considered that changes in RBPs have been associated with various cancers, which could possibly influence the susceptibility to influenza infections (247, 248). With the growing interest in RNA biology, this aspect of IAV infections is likely to receive considerable attention in the future.

In terms of IAV antivirals, the recent progress in determining the structures and mechanisms of the viral polymerase should significantly aid in the current development of drugs aimed at inhibiting different aspects of IAV transcription (249). Through continued progress in defining the fundamental mechanisms that are necessary for IAV infections, replication and intercellular movement, it should become possible to minimize the annual burden caused by IAVs.

## Author Contributions

RD wrote the review with input from DD, RR, HÖ, and HW. DD, RR, HÖ, and HW put together the figures and wrote the figure legends.

## Conflict of Interest Statement

The authors declare that the research was conducted in the absence of any commercial or financial relationships that could be construed as a potential conflict of interest.
